# A database framework for rapid screening of structure-function relationships in PFAS chemistry

**DOI:** 10.1038/s41597-021-00798-x

**Published:** 2021-01-18

**Authors:** An Su, Krishna Rajan

**Affiliations:** Department of Materials Design and Innovation, University at Buffalo, Buffalo, NY USA

**Keywords:** Environmental chemistry, Cheminformatics

## Abstract

This paper describes a database framework that enables one to rapidly explore systematics in structure-function relationships associated with new and emerging PFAS chemistries. The data framework maps high dimensional information associated with the SMILES approach of encoding molecular structure with functionality data including bioactivity and physicochemical property. This ‘PFAS-Map’ is a 3-dimensional unsupervised visualization tool that can automatically classify new PFAS chemistries based on current PFAS classification criteria. We provide examples on how the PFAS-Map can be utilized, including the prediction and estimation of yet unmeasured fundamental physical properties of PFAS chemistries, uncovering hierarchical characteristics in existing classification schemes, and the fusion of data from diverse sources.

## Introduction

Perfluoroalkyl or polyfluoroalkyl substances (PFASs) are compounds that contain at least one fully fluorinated carbon (e.g. -CF_3_, -CF_2_-)^1,2^. With outstanding qualities in chemical and thermal stability, water repellency, and oil repellency, PFASs have been used in a wide range of industrial and commercial products such as food contact materials, ski waxes, fire-fighting foams, water, and stain repellent textiles, medical devices, laboratory supplies, and personal care^1,3^. However, the presence of PFASs in freshwater systems, wildlife, and even human blood^4–6^ have raised serious public concerns about unknown dangers due to PFAS’s high persistence (P), bioaccumulation potential (B), toxicity (T), and ease of being transmitted or transported through the environment^7^. Although legacy PFASs such as perfluorooctanesulfonic acid (PFOS) and perfluorooctanoic acid (PFOA) and some of their precursors are being evaluated to be listed as chemicals of concern and/or considered for regulation^8^, alternate PFASs with similar structures and functionality, such as short-chain perfluoroalkyl carboxylic acids (PFCAs) and perfluoroalkane sulfonic acids (PFSAs), perfluoroalkyl phosphinic acids (PFPiAs), and perfluoroether carboxylic and sulfonic acids (PFECAs and PFESAs), are still being produced and used^8–11^. Recent developments in high-resolution mass spectrometry has made it possible to discover increasing numbers of alternative PFASs which has added thousands of compounds to the PFAS family^12,13^. By May 2020, there were 7,866 structurally-defined compounds under the United States Environmental Protection Agency’s (USEPA) PFAS master list (https://comptox.epa.gov/dashboard/chemical_lists/pfasmaster).

As this family of ‘forever’ compounds grows rapidly, it is nearly impossible to establish hazard data associated with each new PFAS chemistry. Thus, having meaningful classifications of PFAS compounds is extremely important^7,13^. A well-acknowledged PFAS classification system was published in 2011 by Buck *et al*. based on the patterns of chemical structure for each group or subgroup^1^. However, as more and more PFASs have been identified in the past decade, there have been efforts to update the Buck’s classification system. The Organization for Economic Co-operation and Development (OECD) updated the PFAS classification in 2018 by adding new compounds to the family of PFASs such as side-chain aromatics^2^. As the PFAS classification improves and evolves, (e.g. Wang *et al*.^13^ and Sha *et al*.^14^), the present works aims at establishing an automated PFAS classification system that can readily capture the updates in PFAS classification. Machine learning approaches have been used to identify patterns in existing data on PFAS’s properties (including bioactivity, bond strength, and sources) and used to make predictions^14–16^. Most of the machine learning methods in these studies are based on supervised learning using the molecules’ structural information as ‘features’ and properties as ‘labels’; however, the number of PFASs with known properties is significantly lower than the number of PFASs with identified structures^13^. On the other hand, unsupervised learning, an exploratory machine learning technique, capable of finding hidden patterns or grouping in data without the need of any labels^17^, has not been fully utilized in PFAS studies.

In this study, we describe a framework that maps the data on the structure and/or functionality (e.g. bioactivity, physicochemical property) of PFASs and present the structure-function relationship through a 3D visualization schema (PFAS-Map). The first step involves representing each PFAS compound Simplified Molecular Input Line Entry System (SMILES)^18^ format and calculating 1D and 2D molecular descriptors as well as PubChem fingerprints using PaDEL-descriptor^19^ methods to generate the multidimensional features for each compound. As a pre-processing step, these nearly 2,000 features are reduced by principal component analysis (PCA) with more than 70% original information retained. From this feature space, t- Distributed Stochastic Neighbor Embedding (t-SNE) algorithm is applied to visualize the high dimensional space into three dimensions^20^. In parallel, PFASs are automatically classified in classes/subclasses based on their SMILES and molecular descriptors. The SMILES classification results, along with the data on PFAS functionality, are also captured in PFAS-Map. With structures, classification, and functionality all displayed simultaneously, the structure-function relationship of PFASs can be rapidly screened using this PFAS-Map in an organized, straightforward way.

## Results

### Data souces: US EPA PFAS Master List

The US EPA PFAS Master List of PFAS substances (https://comptox.epa.gov/dashboard/chemical_lists/pfasmaster) is a growing inventory that consists of all registered PFASs lists from within and outside the United States Environmental Protection Agency (US EPA), organized and structure-annotated by EPA researchers within the National Center for Computational Toxicology^21^. By May 2020, the number of PFASs included in the list had increased to 7,866. For our study, we removed chemical structures with invalid or non-canonical SMILES as well as duplicate chemical structures generated after preprocessing steps (e.g. removing salts subgroups, deleting isotopic specifications, neutralizing ionic structures), leaving 6,134 distinct chemical structures for further processing.

### Incorporation of structure-function classification

**Fig. 1:**
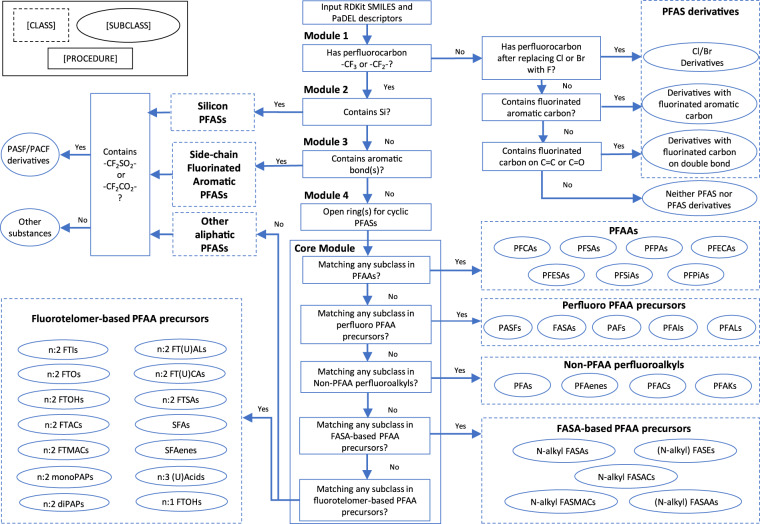
Structure classification of PFASs in PFAS-Map.

The classification of PFAS structure consists of a core module and a series of filtering and transformation modules (Fig. 1). The core modules classify the PFASs that have well-defined classes and subclasses in Buck’s classification system^1^ or OECD’s classification^2^ and its following refinements^13,22^, while the filtering modules classify the rest of the PFASs (see methods for details). PCA reduces ~2,000 descriptors into 74 principal components that capture 70% of explained variance in PFASs’ structure (see “Scree plot” in figshare_File_1). t-SNE visualizes the principal components in a three-dimensional space so that the PFASs presented as three-dimensional arrays are distributed along with the structure classification results that include the PFAS function data. The t-SNE visualization starts by translating distances between data points in the high dimensional space, into a symmetric joint probability that encodes their similarities. Likewise, a similar probability distribution is defined for the low dimensional space which describes the data similarity. The algorithm follows by optimizing the positions in the low dimensional space, in order to minimize the difference between the joint probability distributions^23^. Step and perplexity, the two important hyperparameters for t-SNE^24^, are set to 1,000 and 50, respectively, based on the clustering of PFAS classes/subclasses. Examples of PFAS clustering with different values of hyperparameters are included in the “optimization” folder in figshare_File_1.

### Structure-function database architecture

**Fig. 2:**
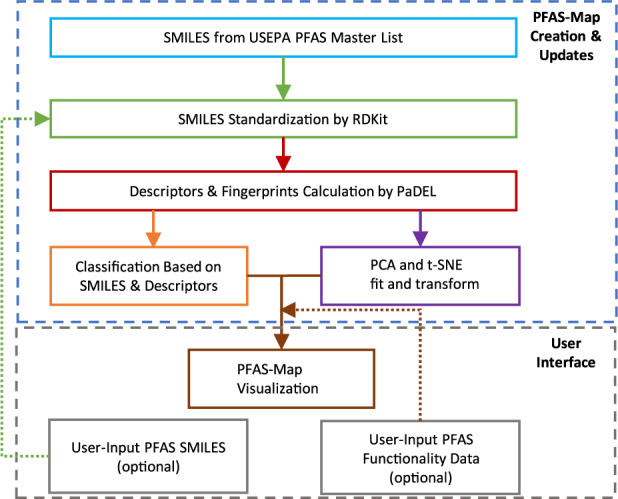
The architecture of PFAS-Map.

The architecture of PFAS-Map is shown in Fig. 2. The key modules of PFAS-Map include SMILES standardization by RDKit (http://rdkit.org), descriptors calculation by PaDEL^19^, PFAS structure classification, PCA and t-SNE training and transformation, and visualization of t-SNE/PCA transformation results and classification results. The PFASs from US EPA PFAS Master List (EPA PFASs) are preprocessed through the framework, and this output serves as the foundation of the PFAS-Map. Based on this foundation, SMILES of PFASs from user input go through the same process including SMILES standardization, descriptors calculation, and classification, except that the descriptors calculated are directly transformed using the PCA model that is trained by EPA PFASs. Meanwhile, the user-input PFAS functionality data can be visualized on PFAS-Map along with the t-SNE/PCA transformation results and classification results.

**Fig. 3:**
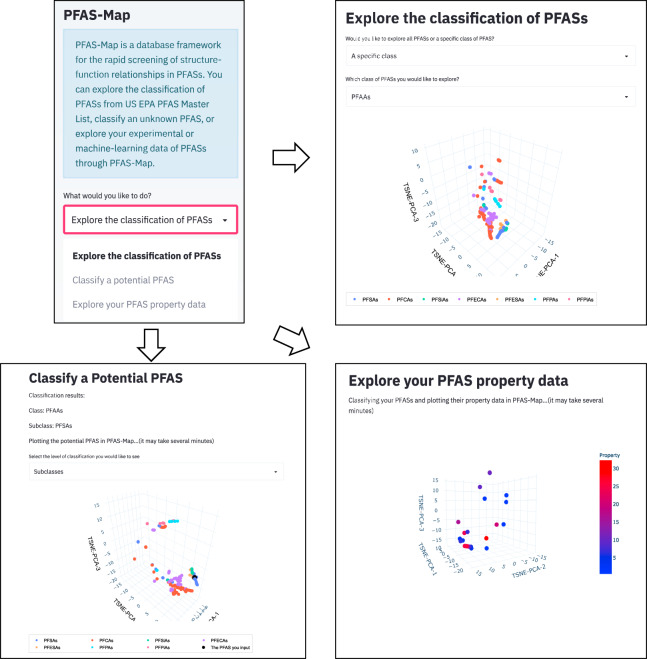
The user interface of PFAS-Map. Upper left: side bar for function selection; Upper right: exploring EPA PFASs; Lower left: classifying potential PFASs; Lower right: exploring user-input PFAS functionality data.

Some of the functionalities of PFAS-Map (Fig. 3) include (i) the ability to query and visualize classification of PFAS chemistry in terms of molecular structure, (ii) explore similarity or dissimilarity of new or existing PFAS from the SMILES code and populate the PFAS-Map with SMILES and/or functionality information of new PFAS, and (iii) readily explore and establish potentially new structure-function relationships.

## Discussion

In this section, we provide some examples of the utility of the PFAS-Map.

**Fig. 4:**
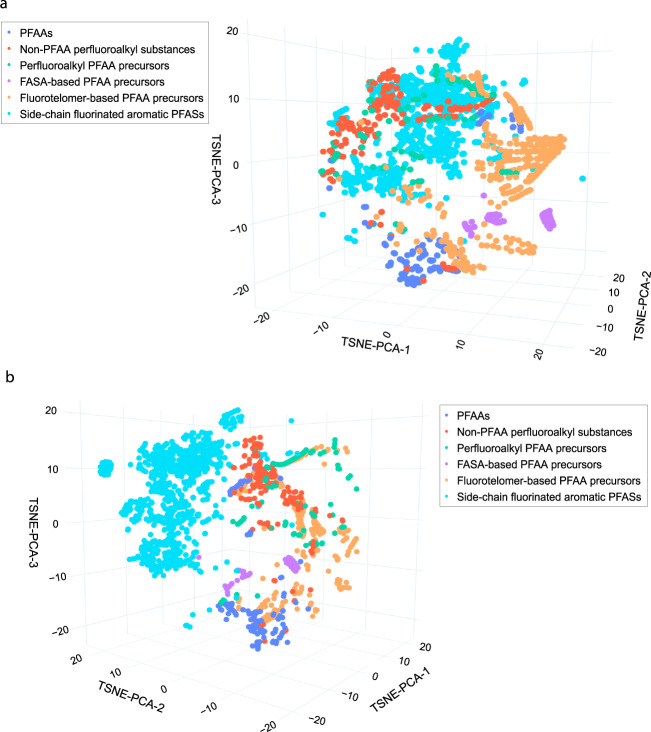
PFAS-Map showing EPA PFASs in classes from two different perspectives. a). The perspective showing the classes of aliphatic PFASs. b) The perspective showing the separation of aromatic PFASs from aliphatic PFASs. Abbreviations: PFAA: perfluoroalkyl acids. An interactive version of this figure is provided in figshare File 1.
  As another example, the subclasses of two well-defined classes, FASA-based PFAA precursors and fluorotelomer-based PFAA precursors, are shown in Figs. 5 and 6, respectively. The subclasses in the class of FASA-based PFAA precursors follows the structural pattern as C_n_F_2n+1_-SO_2_N(C_m_H_2m_)-R^1^. Separation of different subclasses as well as trajectories of behavior can be tracked in the t-SNE-PCA components represented in the 2D projection of PFAS-Map (Fig. 5). First, the perfluoroalkyl chain length increases mainly due to increase in the value of t-SNE-PCA-2. In addition, the sizes of N-alkyl group separate the compounds having the same functional group but different sizes of N-alkyl group. Furthermore, the PFASs with the same perfluoroalkyl chain but different functional groups are also separated. The n:2 fluorotelomer subclasses in the class of FASA-based PFAA precursors follows the structural pattern as C_n_F_2n+1_-C_2_H_4_-R^1^. The distribution pattern of the n:2 fluorotelomers are similar to the FASA-based precursors---the perfluoroalkyl chain length increases mainly along t-SNE-PCA-2 (except fluorotelomer phosphates) while the functional groups separate subclasses. Similar patterns in the perfluoroalkyl chain lengths, size of alkyl group(s), and the separation based on functional groups are also observed in the subclasses of other classes, as is shown in figshare File 1.

- Detection and visualization of sub-classifications of PFAS chemistry.
  Figure 4 shows a clear clustering of aromatic and aliphatic PFAS chemistries (Fig. 4b) with the cluster of aromatic PFAS (light blue) and aliphatic PFAS (mixed colors). In the aliphatic cluster one can observe four sub-clusters---non-PFAA perfluoroalkyls (orange), perfluoroalkyl PFAA precursors (green), PFAAs (dark blue), and FASA-based and fluorotelomer-based precursors (purple and orange) as is shown in Fig. 4a. Hence in PFAS-Map has the capacity to capture established classifications^1,2^ as well as reveal sub-classifications that would not otherwise be easily seen.

**Fig. 5:**
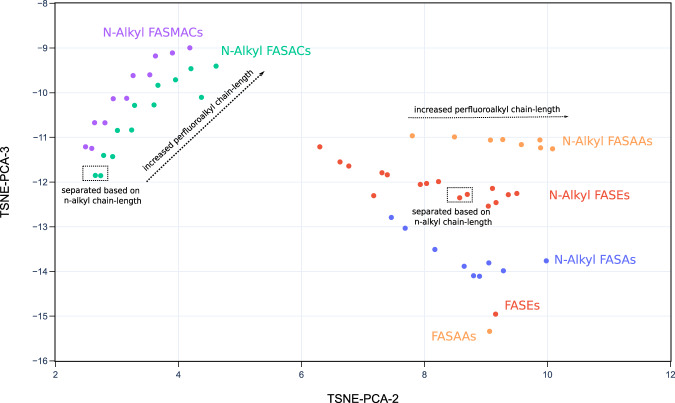
2D projection of PFAS-Map (TSNE-PCA-2 and TSNE-PCA-3) showing all subclasses under the class of FASA-based PFAA precursors. Abbreviations: FASEs: perfluoroalkane sulfonamidoethanols; FASAs–perfluoroalkane sulfonamides; FASAAs: perfluoroalkane sulfonamidoacetic acids; FASACs: perfluoroalkane sulfonamidoethyl acrylates; FASMACs–perfluoroalkane sulfonamidoethyl methacrylates. An interactive version of this figure is provided in figshare File 1.

**Fig. 6:**
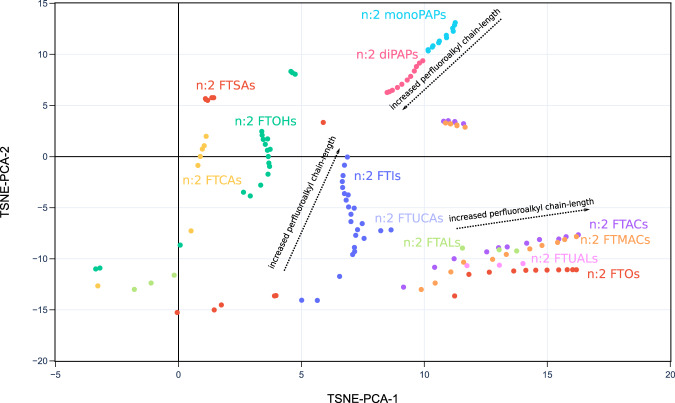
2D projection of PFAS-Map (TSNE-PCA-1 and TSNE-PCA-2) showing n:2 fluorotelomer subclasses under the class of fluorotelomer-based PFAA precursors. Abbreviations: FTOHs: fluorotelomer alcohols; FTACs: fluorotelomer acrylates; FTMACs: fluorotelomer methacrylates; FTIs: fluorotelomer iodides; FTOs: fluorotelomer olefins; FTSAs: fluorotelomer sulfonic acids; monoPAPs: fluorotelomer phosphates, monoester; diPAPs: fluorotelomer phosphates, diester; FTALs: fluorotelomer aldehydes; FTCAs: fluorotelomer carboxylic acids; FTUALs: fluorotelomer unsaturated aldehyde; FTUCAs: fluorotelomer unsaturated carboxylic acid. An interactive version of this figure is provided in figshare File 1.
- Screening the relationship between PFASs structure and toxicity from two sets of experimental data.
  The PFAS-Map helps us visualize trends in experimental data on PFAS’s activity/property relationships so as to uncover hidden structure-toxicity relationships that could not have been easily seen when the same data is presented in tabular form. Weiss *et al*. studied the competition between a series of PFASs and thyroxine (T4) for binding to the human thyroid hormone transport protein (TTR) and they showed the competition in the T4-TTR binding (%) (lower value means a higher amount of PFAS is binding to TTR)^25^. Figure 7 plots the T4-TTR binding data on the 2D projection of PFAS-Map. The binding data shows similar trends in PFCAs and PFSAs: the binding is higher (shown in red) when it comes to short chain-length (C4) or chain-length longer than C10, while the binding is the lowest (shown in blue) when it comes to C8. Hence, it is straightforward to have an estimated range of binding values for C5, C7, C9, C10, C11 for PFSAs, and C5 for PFCAs. Meanwhile, the significantly different binding values seen from the map between 2H-Perfluoro-2-octenoic acid (FTUA (6:2)) and 6:2 fluorotelomer alcohol (FTOH (6:2)) and the high binding value for FOSAs and FOSEs infers that the T4 competition exists mostly in PFAAs but rarely in PFAA precursors.

**Fig. 7:**
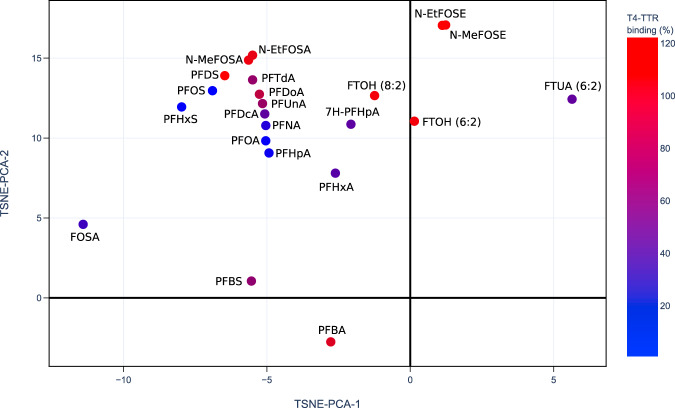
PFAS competed T4-TTR binding (%)^25^ data shown on the 2D projection (TSNE-PCA-1/TSNE-PCA-2) of the PFAS-Map. Abbreviations: PFBA: perfluorobutanoic acid; PFBS: perfluorobutane sulfonic acid; PFHxA: perfluorohexanoic acid; 7H-PFHpA: 7H-perfluoroheptanoic acid; PFHpA: perfluoroheptanoic acid; PFHxS: perfluorohexane sulfonic acid; PFOA: perfluorooctanoic acid; PFNA: perfluorononanoic acid; FOSA: perfluorooctanesulfonamide; PFOS: perfluorooctanesulfonic acid; PFDcA: perfluorodecanoic acid; PFUnA: perfluoroundecanoic acid; PFDS: perfluorodecane sulfonic acid; PFDoA: perfluorododecanoic acid; PFTdA: perfluorotetradecanoic acid; FTUA (6:2): 2H-perfluoro-2-octenoic acid; N-MeFOSA: N-methylperfluorooctanesulfonamide; FTOH (8:2): 8:2 fluorotelomer alcohol; FTOH (6:2): 6:2 fluorotelomer alcohol; N-EtFOSA: N-ethylperfluorooctanesulfonamide; N-MeFOSE: N-methyl-N-(2-hydroxyethyl)perfluorooctanesulfonamide; N-EtFOSE: N-ethyl-N-(2-hydroxyethyl)perfluorooctanesulfonamide. An interactive version of this figure is provided in figshare File 1.
  The US EPA’s CompTox Chemical Dashboard provides a chemical activity summary for each of the PFASs that have been tested by ToxCast assays^21,26^, and the summaries are visualized in PFAS-Map (Fig. 8). For each PFAS, its chemical activity is characterized by a ‘hit ratio’ (the ratio of the number of active assays to the number of all assays tested^27^). Two significant phenomena are observed. First, most of the compounds with higher hit ratio are PFAAs, and an increased hit ratio is observed for PFAAs as the perfluoroalkyl chain length increases. In addition, the hit ratio is generally lower for the non-acid PFAA precursors. By comparing the results from Figs. 7 and 8, we can find similarities in the structure-toxicity relationship of PFASs. For example, as one of the earliest regulated PFASs, PFOS has the most significant toxicity---it leads to one of the lowest T4-TTR protein bindings (Fig. 7) and, notably, has one of the highest hit ratios (Fig. 8). Also, the non-acid fluorotelomers are generally less toxic than PFAAs based on their higher T4-TTR bindings (Fig. 7) and lower hit ratio (Fig. 8), suggesting that the removal of acidic groups can potentially lower the toxicity of PFASs.

**Fig. 8:**
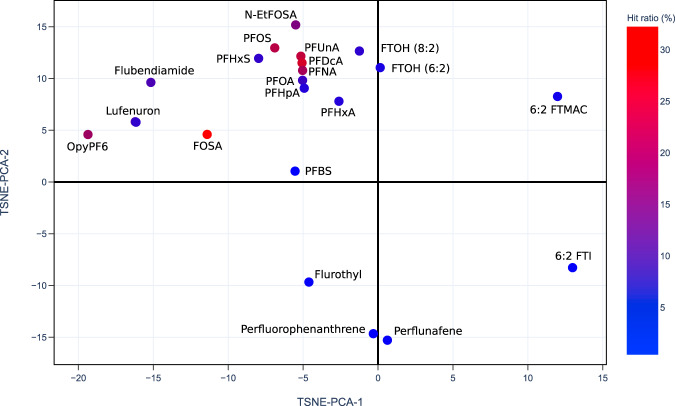
Currently available PFASs ToxCast chemical activity summary data^21,26^ shown on the 2D projection (TSNE-PCA-1 and TSNE-PCA-2) of the PFAS-Map. The hit ratio (the ratio of active assays to the number of all assays tested^27^) in fractional form is converted to percentage (e.g. 210/851 = 24.7% for PFUnA). Abbreviations: PFBS: perfluorobutane sulfonic acid; PFHxA: perfluorohexanoic acid; PFHpA: perfluoroheptanoic acid; PFHxS: perfluorohexane sulfonic acid; PFOA: perfluorooctanoic acid; PFNA: perfluorononanoic acid; FOSA: perfluorooctanesulfonamide; PFOS: perfluorooctanesulfonic acid; PFDcA: perfluorodecanoic acid; PFUnA: perfluoroundecanoic acid; FTOH (8:2): 8:2 fluorotelomer alcohol; FTOH (6:2): 6:2 fluorotelomer alcohol; N-EtFOSA: N-ethylperfluorooctanesulfonamide; OpyPF6: 1-methyl-3-octylimidazolium hexafluorophosphate; 6:2 FTMAC: 6:2 fluorotelomer methacrylate; 6:2 FTI: 1H,1H,2H,2H-perfluorooctyl iodide. An interactive version of this figure is provided in figshare File 1.
- Screening structure-activity relationships of PFAS chemicals.

**Fig. 9:**
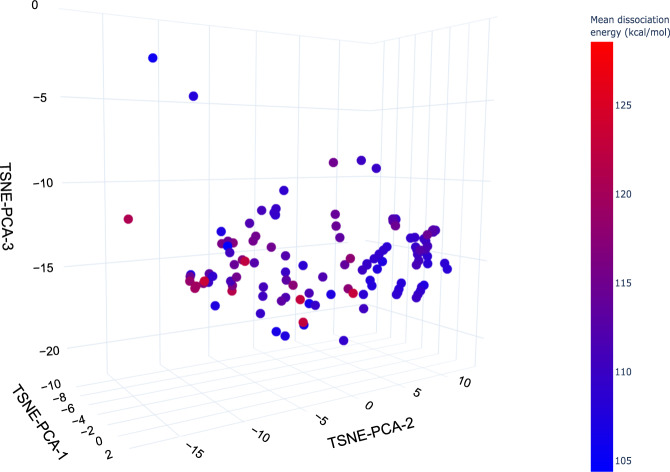
PFAS-Map showing the predicted mean C-F bond dissociation energy from the Raza *et al*.’s work “A Machine Learning Approach for Predicting Defluorination of Per and Polyfluoroalkyl Substances (PFAS) for Their Efficient Treatment and Removal”^15^. An interactive version of this figure is provided in figshare File 1.

PFAS-Map can also be coupled with dissociation data to study the structure-persistence relationship of PFASs. Figure 9 shows the mean C-F bond dissociation energy (the average of all C-F bonds’ dissociation energy in a molecule) calculated based on Raza *et al*.’s work on machine learning prediction of PFAS defluorination^15^. The PFAS map highlights the trend that the mean dissociation energy generally decreases as the length of perfluoroalkyl chain increases, and also that the mean dissociation energy for aromatic PFASs is significantly higher than those aliphatic PFASs with a similar number of carbons.

The examples discussed above demonstrate the versatility of the PFAS Map. The automated capabilities in our database infrastructure, driven by unsupervised learning methods, provides one the means to easily visualize classification patterns and trends in structures-function relationships in PFAS chemistry. One of the current bottlenecks of PFAS research is the significantly larger number of PFASs with known chemical structures compared to the number of PFASs with known properties. Hence, an unsupervised learning model like PFAS-Map fills a pressing need to appropriately classify most of PFAS molecules which are, at present, unlabeled in terms of their toxicity/hazard impact. Since the PFAS-Map is built using open-source information, it can accommodate updates from the scientific literature on the PFAS classification rules; these changes can be added to the source code of classification program ensuring that new classification patterns are readily tracked. Finally, as noted at the outset of our manuscript, this paper focuses on unsupervised structural classification of PFAS compounds. The PFAS-Map serves as an inference tool to assess the potential functionality of new PFAS molecules when compared with available property data. A clear next stage of development for the PFAS Map is to extend its capabilities to prediction. Aside from applying enhanced machine learning strategies, developing robust predictive methods on toxicity requires the incorporation of additional descriptors that capture the details of molecular mechanisms that govern the interaction of PFAS with biological macromolecules^28,29^, that govern behavior such as bioactivity and bioaccumulation. This will be the subject of forthcoming papers.

## Methods

### SMILES standardization

The motivation for SMILES standardization is that one chemical structure can have various valid canonical SMILES generated by different computational tools or used by different databases. For example, perfluorooctanesulfonic acid (PFOS) has at least three canonical SMILES: C(C(C(C(C(F)(F)S(=O)(=O)O)(F)F)(F)F)(F)F)(C(C(C(F)(F)F)(F)F)(F)F)(F)F (PubChem), OS(=O)(=O)C(F)(F)C(F)(F)C(F)(F)C(F)(F)C(F)(F)C(F)(F)C(F)(F)C(F)(F)F (EPA CompTox), and O=S(=O)(O)C(F)(F)C(F)(F)C(F)(F)C(F)(F)C(F)(F)C(F)(F)C(F)(F)C(F)(F)F (RDKit). Hence, our standardization tool based on RDKit is implemented to convert SMILES from different sources into RDKit SMILES so that a RDKit-SMILES-based PFASs classification algorithm can be designed. User input SMILES goes through SMILES standardization, descriptors calculation, PFAS classification in the same way as EPA PFASs. The only difference is that the descriptors of user input PFAS will be directly transformed by the PCA model pre-trained by the EPA PFASs so that the user input PFAS and EPA PFASs can be shown in the same PFAS-Map.

### Descriptors calculation

The molecular descriptors and fingerprints of the chemical structures are calculated by PaDELPy (https://github.com/ECRL/PaDELPy), a python library for the PaDEL-descriptors software^19^. 1D and 2D molecular descriptors and PubChem fingerprints (altogether called “descriptors” in the following text) are calculated for each chemical structure. The descriptors that have invalid value for a significant number of chemical structures are removed. Simple-count descriptors (e.g. number of C, H, O, N, P, S, and F, number of aromatic atoms) are used for the classification model along with SMILES. Meanwhile, all the descriptors of EPA PFASs are used as training data for PCA.

### PFAS structure classification

As is shown in Fig. 1, module 1 filters the chemical structures not matching the most current definition of PFAS---containing “at least one -CF_3_ or -CF_2_- group”^1,2^. The module categorizes the unmatched chemical structures as “PFAS derivatives” if they fall into any of three subclasses: PFASs having -F substituted by -Cl or -Br, PFASs containing a fluorinated C = C carbon or C = O carbon, or PFASs containing fluorinated aromatic carbons. Otherwise, the chemical structure is marked as “not PFAS”. Module 2 separates the PFASs that contain one or more Silicon atom and classify them as “Silicon PFASs” as no existing rule is available in the literature so far that can further classify the PFASs containing Silicon to our knowledge. After Module 3 filtering the side-chain fluorinated aromatics PFASs defined by OECD^2^, the cyclic aliphatic PFASs are transformed to acyclic aliphatic PFASs in Module 4 by breaking the rings and add a F atom to the beginning and ending carbons of the ring. For example, O=S(=O)(O)C1(F)C(F)(F)C(F)(F)C(F)(F)C(F)(F)C1(F)F (undecafluorocyclohexanesulfonic acid) is converted to O=S(=O)(O)C(F)(F)C(F)(F)C(F)(F)C(F)(F)C(F)(F)C(F)(F)F) (perfluorohexanesulfonic acid). After going through the pre-screen modules, the chemical structures that have not been categorized enter the core module of the classification system. The core module follows a “class-subclass” two-level classification, inheriting the majority of Buck’s classification rules^1^ for the classes including perfluoroalkyl acids (PFAAs), perfluoroalkyl PFAA precursors, perfluoroalkane-sulfonamide-based (FASA-based) PFAA precursors, and fluorotelomer-based PFAA precursors. Additional classes not in Buck’s system but OECD’s classification^2^ and following refinements^13,22^, such as perfluorinated alkanes, alkenes, alcohols, ketones, are also included as the class of non-PFAA perfluoroalkyls. In the core module, the chemical structures are tested to see if they match the structure pattern of each subclass based on their SMILES and molecular descriptors. Detailed classification algorithms can be referred in the source code.

### Principal component analysis (PCA)

A PCA model is trained with the descriptors data of EPA PFASs using Scikit-learn^30^, a Python machine learning module. The trained PCA model reduced the dimensionality of the descriptors from 2090 to fewer than 100 but still obtains a significant percentage (e.g. 70%) of explained variance of PFAS structure. This feature reduction is needed to fasten the computation and suppress the noise in the further processing of the t-SNE algorithm^20^. The trained PCA model is also used to transform the descriptors from user-input SMILES of PFASs so that the user-input PFASs can be included in PFAS-Maps along with the EPA PFASs.

### t-Distributed stochastic neighbor embedding (t-SNE)

The PCA-reduced data in PFAS structure is feed into a t-SNE model, projecting the EPA PFASs into a three-dimensional space. t-SNE is a dimensionality reduction algorithm that is often used to visualize high-dimensionality datasets in a lower-dimensional space^20^. Step and perplexity are the two important hyperparameters for t-SNE. Step is the number of iterations needed for the model to reach a stable configuration^24^, while perplexity defines the local information entropy that determines the size of neighborhoods in clustering^23^. In our study, the t-SNE model is implemented in Scikit-learn^30^. The two hyperparameters are optimized based on the ranges suggested by Scikit-learn (https://scikit-learn.org/stable/modules/generated/sklearn.manifold.TSNE.html) as well as the observation of PFAS class/subclass clustering. A step or perplexity lower than the optimized number leads to a more scattered clustering of PFASs, while a higher value of step or perplexity does not significantly change the clustering but increases the cost of computational resources. Details of the implementation can be found in the provided source code.

### Framework visualization

Combining the classification results with the t-SNE/PCA results, PFASs are visualized in a 3D interactive graph by Plotly (https://plotly.com) with the value of the three components (TSNE-PCAs) as the three coordinates (x, y, z) of the markers, while the colors of markers show the respective class/subclass of the PFASs. For user-input PFAS activity/property data, the data is reflected in the color of the markers or as hover text above the markers.

## Data Availability

The authors declare that the main data supporting the finding of this study are available within the article. All the supporting data have been deposited at figshare^31^. 3D-interactive figures of PFAS-Maps, including the classifications of EPA PFASs and PFAS structure-function relationship screening, are included in the “figshare File 1” folder. The datasets required for the operation of the PFAS-Map (e.g. the SMILES, t-SNE/PCA results, and classification results of EPA PFASs) are included in the “PFAS-Map” folder. The data is also available on the Materials Data Engineering Laboratory - MaDE@UB portal (http://madeatub.buffalo.edu).

## Code availability

The code supporting the finding of this study have been deposited at figshare^31^. All code needed for the user interface of PFAS, as well as the repeating of data pre-processing are included in “PFAS-Map” folder. The folder also contains a detailed PDF instruction and a demonstration video for the installation and use of the PFAS-Map. The code is also available on the Materials Data Engineering Laboratory - MaDE@UB portal (http://madeatub.buffalo.edu).
